# Vitamin B12 Deficiency Resembling Acute Leukemia: A Case Report

**DOI:** 10.31729/jnma.6600

**Published:** 2021-11-30

**Authors:** Nisha Sharma, Saru Kunwar, Anil Kumar Shrestha

**Affiliations:** 1 Kanti Children's Hospital, Maharajganj, Kathmandu, Nepal; 2 Department of Pediatrics, Kanti Children's Hospital, Maharajganj, Kathmandu, Nepal

**Keywords:** *anemia*, *leukemia*, *pancytopenia*, *vitamin B12 deficiency*

## Abstract

Vitamin B12 deficiency in children can cause megaloblastic anemia, poor growth, and increased chances of infections. It is an important reversible cause of bone marrow suppression which at the time of presentation can mimic hematological malignancy. Therefore, it should be considered as a differential diagnosis in cases suspected of acute leukemia. We report a case of 14 months old child who had atypical presentation of vitamin B12 deficiency. He had chronic fever, decreased feeding and increased paleness for one year. Pancytopenia with severe anemia was present along with 19% reactive/atypical cells in peripheral blood smear suggesting acute leukemia. However, bone marrow aspiration and biopsy showed features of megaloblastic anemia. Vitamin B12 level measured was very low and treatment with cyanocobalamin caused drastic improvement in the child's condition.

## INTRODUCTION

Vitamin B12 is an essential micronutrient especially during infancy and early childhood. Children with its deficiency usually present with nonspecific symptoms like irritability, weakness, developmental delay and failure to thrive.^1^ Vitamin B12 is an important cofactor in cell metabolism and DNA synthesis.^2^ Its deficiency increases erythroblast apoptosis resulting in anemia or even pancytopenia that resembles hematologic malignancy.^3^ Other findings include increased Mean Corpuscular Volume (MCV), hypersegmentation of neutrophils, reticulocytosis, leukopenia or leukocytosis and thrombocytopenia.^4^

We present a case of 14 months old child whose features were initially suggestive of acute leukemia but was later diagnosed as vitamin B12 deficiency.

## CASE REPORT

A 14 months old boy was brought to our center with chief complaints of recurrent episodes of fever for one year, paleness of skin and decreased appetite since six months. Each febrile episode lasted for around one week with temperature up to 104°F, relieved following medication but reoccurred after one month. Paleness of body was present despite absence of bruises, petechial rashes or bleeding from any orifices. Child was born via non-consanguineous marriage, delivered normally at hospital with birth weight of 3700grams and was up to date with immunizations. He was fed exclusively on breast milk for up to six months of age and though supplementary diet was started, he fed mostly on mother's milk. He was developmentally normal but according to mother, for the past three months his activities had markedly decreased. There was no history of known blood illnesses or malignancies in family. Since the family had low socioeconomic status, animal products were less consumed and mother took mostly vegetarian diet.

On examination, the child was weak, irritable, pale, mildly icteric and afebrile with bilateral non-pitting pedal edema. Saturation was 80% in room air but child was not in respiratory distress. Multiple aphthous ulcers were present over palate and mucosa. Lymph nodes were not palpable. Liver was just palpable but the spleen was not. Rest of the examination findings was normal.

He had pancytopenia with severe anemia, hemoglobin 5.6gm%, MCV 109fl, MCH 36.1pg, MCHC 33.1%, WBC count 3,300/mm^3^ and platelets count 75,000/mm^3^.

In peripheral blood smear, leucoerythroblastic blood picture was present with anisocytosis, spherocytosis and 2% schistocytes. There were 19% reactive/atypical lymphocytes and platelets were reduced to 6-7/hpf. Corrected reticulocytes count was 0.4%. LDH level was high 4265U/L. Bilirubin was elevated to 3.74mg/dl with direct bilirubin 0.58mg/dl but rest of the liver function tests were normal. Direct Coomb's test was negative. Renal function tests, iron profile and screening test for G6PD deficiency were normal.

Acute leukemia was suspected. Bone marrow aspiration and biopsy was done. Packed RBC was transfused. Meanwhile, vitamin B12 and folic acid level reports revealed their values12.5pg/ml (normal 200-1100pg/ml) and 10.9ng/dl (normal5.21-10ng/dl) respectively.

Bone marrow aspiration showed mildly hypocellular picture with 37% nucleated cells, 55% dyserythropoetic cells and 10% ringed sideroblasts. Normal leukopoiesis with 21% myelocyte, 9% metamyelocyte, 15% neutrophil and 1% eosinophil were seen.

Bone marrow trephine biopsy showed limited marrow with megaloblastic changes.

Mother's vitamin B12 level was also very low 12.5pg/ml but folic acid level was normal. Since our patient fed primarily on mothers' milk with minimal supplementary diet, final diagnosis of megaloblastic anemia secondary to vitamin B12 deficiency due to dietary insufficiency was made.

**Figure 1:**
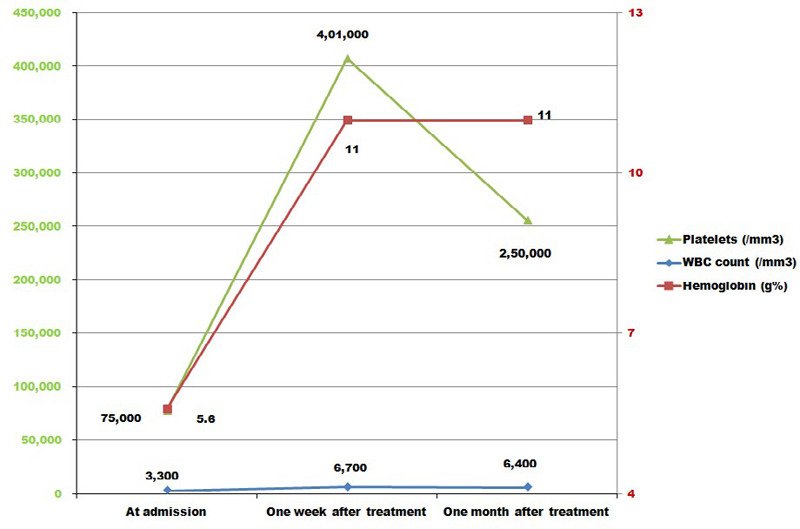
Levels of hemoglobin, WBC, platelet count before and after treatment.

Treatment was done with vitamin B12 supplementation along with folic acid. Injection cyanocobalamin 500mcg was given intramuscularly daily for seven days followed by weekly for 1 month. There was marked improvement in child's physical activity. Hematological changes are graphed (Figure 1).

Other blood parameters were reassessed, results of which can be appreciated (Table 1).

**Table 1:** Changes in blood parameters before and one month after treatment.

Time	Vitamin B12 (pg/ml)	LDH (IU/L)	Bilirubin total (mg/dl)	MCV (fl)	Retics (%)	Peripheral Blood Smear		
						RBC	WBC	Platelets
At admission	12.5	4265	3.74	109	0.4	Anisocytosis, spherocytosis and 2% schistocytes	19% reactive lymphocytes	Reduced (6-7/hpf)
						Leukoerythroblastic blood picture		
One month after treatment	983	280	0.8	85	0.5	Normocytic, normochromic	No atypical cells or blast cells	Adequate
						Normal blood picture		

## DISCUSSION

Pancytopenia is not a frequent presentation of vitamin B12 deficiency.^5^ A study in 279 pancytopenic children, revealed that the most common etiologies were acute leukemia in 32.2% of patients, aplastic anemia in 30.8% and megaloblastic anemia in 13.2% of patients.^6^A study conducted by E Andres et al. showed that hematological abnormalities in vitamin B12 deficiency manifested in only two-thirds of the individuals and severe manifestations were present in only 10% of the patients which included: symptomatic pancytopenia in 5%, 'pseudo' thrombotic microangiopathy in 2.5%, severe anemia (Hb levels <6g/dl) in 2.5% and hemolytic anemia in 1.5%.^5^

Our patient had severe symptomatic macrocytic anemia, pancytopenia, hemolytic anemia and features suggestive of pseudo-thrombotic microangiopathy (TMA) (thrombocytopenia, very high LDH levels, a low reticulocyte count strongly suggest pseudo-TMA and cobalamin deficiency as possible underlying cause).^7^

Vitamin B12 deficiency concomitant with leukemia have been reported in few cases and there have been challenges in distinguishing between them.^8,9^ In our case, along with the above findings, peripheral blood smear examination showed 19% reactive lymphocytes with leukoerythroblastic picture suggesting acute leukemia. Bone marrow examination can be a very useful tool to determine the etiology of pancytopenia and come to a diagnosis,^6^ which in our case revealed mildly hypocellular marrow with dyserythropoiesis and megaloblastic changes. It was supported by low serum vitamin B12 level.

Vitamin B12 deficiency is rare in infancy. However, most cases are seen in exclusively breastfed infants as a result of maternal insufficiency.^10^ Women in low-income countries, including up to one-third in rural Nepal have been found to have depleted vitamin B12 levels.^11^ In our case mother's vitamin B12 level was also low. Since she followed vegetarian diet most of her life, her likely cause of vitamin B12 deficiency was dietary insufficiency. Due to financial constrain, further investigations to rule out other causes could not be done.

Early diagnosis and treatment of vitamin B12 deficiency with its replacement reverses the clinical signs and improves outcome in a child. Since vitamin B12 supplementation is done for months, patient should be regularly followed up. Deranged parameters should be rechecked and treatment dose should be adjusted accordingly.
